# Compliance and enforcement of a partial smoking ban in Lisbon taxis: an exploratory cross-sectional study

**DOI:** 10.1186/1471-2458-13-134

**Published:** 2013-02-14

**Authors:** Sofia B Ravara, Miguel Castelo-Branco, Pedro Aguiar, Jose M Calheiros

**Affiliations:** 1 Health Sciences Research Centre, Faculty of Health Sciences, University of Beira Interior, Av. Infante D. Henrique, 6200-506 Covilhã, Portugal; 2 National Institute of Preventive Cardiology Professor F. Pádua, R. Doutor Nicolau Bettencourt 45, 1050-078 Lisbon, Portugal; 3 National School of Public Health, New University of Lisbon, Av. Padre Cruz, 1600-560, Lisbon, Portugal; 4 National Institute of Health Dr. Ricardo Jorge, Av. Padre Cruz, 1649-016, Lisbon, Portugal

**Keywords:** Tobacco control, Second-hand smoke, Smoking partial ban, Smoke-free policy, Compliance, Enforcement, Taxi, Smoke-free cars

## Abstract

**Background:**

Research evaluating enforcement and compliance with smoking partial bans is rather scarce, especially in countries with relative weak tobacco control policies, such as Portugal. There is also scarce evidence on specific high risk groups such as vehicle workers. In January 2008, Portugal implemented a partial ban, followed by poor enforcement. The purpose of this study was to explore the effectiveness of a partial smoking ban in a pro-smoking environment, specifically transportation by taxi in the city of Lisbon. Ban effectiveness was generally defined by ban awareness and support, compliance and enforcement.

**Methods:**

Exploratory cross-sectional study; purposive sampling in selected Lisbon streets. Structured interviews were conducted by trained researchers while using taxi services (January 2009-December 2010). Participants: 250 taxi drivers (98.8% participation rate). Chi-square, McNemar, Man Whitney tests and multiple logistic regression were performed.

**Results:**

Of the participants, 249 were male; median age was 53.0 years; 43.6% were current smokers. Most participants (82.8%) approved comprehensive bans; 84.8% reported that clients still asked to smoke in their taxis; 16.8% allowed clients to smoke. Prior to the ban this value was 76.9% (p < 0.001). The major reason for not allowing smoking was the legal ban and associated fines (71.2%). Of the smokers, 66.1% admitted smoking in their taxi. Stale smoke smells were detected in 37.6% of the cars. None of the taxi drivers did ever receive a fine for non-compliance. Heavy smoking, night-shift and allowing smoking prior the ban predicted non-compliance.

**Conclusions:**

Despite the strong ban support observed, high smoking prevalence and poor enforcement contribute to low compliance. The findings also suggest low compliance among night-shift and vehicle workers. This study clearly demonstrates that a partial and poorly-enforced ban is vulnerable to breaches, and highlights the need for clear and strong policies.

## Background

Worldwide, tobacco remains a leading preventable cause of death, killing nearly 6 million people each year, including more than 600,000 who had been exposed to second-hand smoke (SHS) [1]. Enacting 100% smoke-free policies (SFPs) is the only effective way to eliminate SHS and its consequences [2]. Examples from countries where comprehensive SFPs have been adopted, with few exemptions and no allowance for smoking areas in hospitality venues, teach us that good compliance and self-regulation can be achieved [1-6]. Good smoke-free policies become self-enforced over time, relying on self-regulation and changes in social norms. These changes occur when comprehensive SFPs are fully implemented, while media campaigns and ongoing community education efforts promote public awareness and support. In addition, planned implementation, strong and comprehensive enforcement and monitoring over time, especially during the first years after bans come into force, are critical to achieving public acceptance and compliance, self-regulation and social norm change [2-9]. This may be particularly needed in “Mediterranean tolerant” countries and in pro-smoking environments [10-12]. While smoking bans have raised awareness of SHS exposure in many public places, this may be not the case in particular enclosed settings such as motor vehicles. Smoking in cars and motor vehicles is particularly harmful, due to dangerously high concentration of pollutants in these confined enclosed spaces [13-16]. Furthermore, smoking in cars is also a risk factor for traffic accidents [17]. Studies evaluating the effectiveness of comprehensive SFPs are common, but research evaluating enforcement and compliance with partial bans is rather scarce, especially in countries with relative weak tobacco control policies, such as Portugal. There is also scarce evidence that specific high risk groups may comply less with the ban. Although many European countries have ratified the Framework Convention on Tobacco Control, tobacco control progress in Europe has been disappointingly slow [1,18]. There is, therefore, a need for background research evaluating the impact of partial bans. In January 2008, Portugal implemented a partial ban, full of ambiguities and exemptions, followed by poor enforcement [12,19,20]. Smoking areas are allowed in hospitality venues and shopping malls and many exemptions are accepted. Patchy compliance has been reported and smoke-free hospitality venues are fast changing into smoking areas due to:

• poor enforcement [19,21,22].

• low awareness of second-hand smoke hazards [12,23].

• the general impact of the ongoing economic crisis and fear among business owners of losing customers [21,22].

Nevertheless, few independent studies are currently evaluating compliance with the ban in Portugal. Past research predicted low compliance in high smoking prevalence and pro-smoking environments [10,12]. We hypothesized that a partial and poorly-enforced ban would have limited power to generate public awareness and self-regulation, resulting in poor compliance over time, especially in pro-smoking environments. Recent studies have identified breaches of the smoking ban in public transports and work vehicles, and high smoking prevalence among taxi drivers [24,25]. Few studies have assessed compliance and enforcement with smoking bans in taxis or other work vehicles, or factors associated with this behaviour. Under current Portuguese law, smoking is banned in workplaces and public transports. The purpose of this study was to explore the effectiveness of the Portuguese partial ban in a pro-smoking environment, specifically public transportation by taxi in the city of Lisbon. We hypothesized that both taxi users and taxi drivers might see taxis as less public than other public places and that enforcement would be both more difficult and less frequent. Effectiveness of the ban was generally defined by ban awareness and support; compliance and enforcement. We also aimed to identify factors associated with ban support and compliance.

## Methods

### Study design, site, study population and sampling

An exploratory cross-sectional study was carried out using interviewer-administered questionnaires and direct observation. Research methods had to take into account a limited budget and time constraints. During the study period there were 3.450 taxi licenses in Lisbon. Our study population was taxi drivers. Systematic direct observation evaluated the display of the required smoking ban signs in taxis and direct and indirect signs of tobacco consumption. Face-to-face structured interviews were conducted between January 2009 and December 2010 by trained researchers while using taxi services in Lisbon city. A previous study had highlighted the difficulty of obtaining a random sample of vehicles in a busy city [24]. Considering the study aims and this limitation, a purposive sampling was applied. We sought to include circulating taxis arriving from different places of Lisbon. We selected a subset of 20 streets in Lisbon centre, accordingly to the geographic area, using a map of the main streets of Lisbon centre (5 in each main directional axis of the city: North, South, East, and West). These were main and busy traffic streets that intersect the city axes. Circulating taxis travelling along these streets are not local traffic; instead they arrive from all parts of the city. Taxi drivers were only paid the taxi fare, independently of their acceptance to collaborate on the survey. The procedure for approaching the taxis on the streets was a systematic way, i.e. the interviewers systematically stopped the first taxi available that was circulating on the selected streets, until they reached their quota for that day, accordingly to the city area and time sampling frame. We excluded taxis waiting at taxis ranks. After stopping the taxi, interviewers indicated the driver their destination as “opportunistic natural clients”. If the taxi driver would refuse to be interviewed, researchers would complete their taxi ride until they had reached their destination, as had been previously settled. For time sampling, we assumed that weather and other seasonal conditions, and also work-schedule shifts, might influence the study’s outcomes. Field work was therefore conducted between 7 a.m. and 3 a.m. and during all seasons of the year. We calculated a sample frame by randomly selecting days of the week, weeks and work-schedule shifts. We aimed to conduct the study as naturally as possible to minimise bias, especially social desirability bias. We also wanted to maximise empathy and taxi driver collaboration. With these aims in mind, we used pen and paper to collect field data. Recruitment was halted when no new relevant information could be elicited [26]. The final sample included 250 taxi drivers (98.8% participation rate): 249 male, median age 53.0 years. Of those 3 taxi drivers that refused to participate, signs of tobacco consumption were observed in only one taxi. The questionnaire was based on 30 initial semi-structured interviews, using a list of specific questions and topics to be discussed. This preliminary field work was conducted by the main researchers who also analysed together and categorised the contents as pre-coded answers. To ensure feasibility and flexibility, and to add verbatim quotation, the final questionnaire comprised a combination of 18 closed-ended and 4 open-ended questions. Spontaneous comments were systematically recorded by the researchers and analysed as verbatim quotations. Field testing was performed with a small sample of 10 taxis. On average, the interviews lasted 15 to 20 minutes. All participants gave their verbal consent to collaborate, after the researchers had explained the study aims, the institutions involved, and warranted anonymity. The study protocol was approved by the University of Beira Interior Research Ethics Committee.

### Questionnaire, measures and outcomes

The questionnaire contained standard items on socio-demographics and work-shift schedule, and taxi sharing with colleagues. Questions on smoking behaviour included smoking status and smoking behaviour inside the taxi (colleague included), tobacco consumption (cigarettes per day), willingness to quit and cessation support. The questions “have you ever smoked?”, “do you currently smoke?”, and “when did you quit?” were used to access smoking status. Participants were classified as never-smokers, smokers, and ex-smokers (having quit more than 1 year ago). Participants were also asked if they had ever attended a session addressing the ban (including SHS health hazards, and those associated with smoking in vehicles) or cessation support. Additionally, the questionnaire addressed SFP awareness and support; ban compliance and enforcement.

### SFP awareness and support

SFP awareness was accessed by observing the display of required smoking ban signs and by two open-ended questions to address the understanding of SFPs or SHS beliefs:

• Do you know the main reasons for smoking restrictions in indoor public spaces, including public transportation?

• Do you allow clients to smoke in your taxi? If not, what are the major reasons? These two questions were asked twice to access behaviour before and after the smoking ban came into force.

We also asked about smoking restrictions in the driver’s home and car.

SFP support was evaluated by asking:

• Do you agree with total smoking bans in any indoor public spaces (public buildings, workplaces, restaurants, cafes, shops, etc., and public transport including taxis)?

This item was accessed using a 5-point Likert scale ranging from “strongly disagree” (1) to “strongly agree” (5). For data analysis, ban support was calculated aggregating “agree” and “strongly agree”, and strong ban agreement included only “strongly agree”.

### Ban compliance and enforcement

Ban compliance was evaluated by self-reported compliance and direct and indirect signs of tobacco consumption such as observing smoking in the taxi and detection of smoke smell, cigarette butts or ash. Self-reported compliance was analysed in two ways:

• Compliance: drivers report that they do not smoke in the taxi.

• Self-enforcement: drivers report that they do not allow clients smoke in the taxi.

We also inquired about clients’ compliance, i.e. if clients ask to smoke in the taxi.

Questions: Do clients ask you to smoke in the taxi? If they ask you what is your answer? Do you smoke in the taxi?

Enforcement was evaluated by self-reported fine application or overhaul.

### Data analyses

Data was analysed using the SPSS statistical package (version 17). Descriptive analysis was performed by calculating the absolute and relative frequencies and the crude odds ratio (OR). Bivariable analyses were conducted using chi-square tests to compare categorical variables, and t tests, or Mann–Whitney tests, to compare means or medians. McNemar tests were used to compare matched samples. A two-sided p value of 0.05 was considered statistically significant. Multiple logistic regression analysis (MLR) was performed to identify factors associated with ban support, self-enforcement and compliance. Self-enforcement and compliance were coded as 1 (yes) and 0 (no). Strong ban agreement was the dependent variable in MLR ban support: stronger agreement (1), versus less or no agreement (0). MLR independent variables included those already identified in the literature [3,10,12,13,27] or significantly associated with the dependent variables in the bivarible analysis, such as: age-group, smoking status, tobacco consumption, shift work, ban support, SHS beliefs (reporting health protection as the main reason for the ban), private smoking restrictions (home and car) and participants’ norms prior the ban (allowing clients smoking in the taxi prior the ban). A backward elimination of explanatory variables at a 5% level of significance was performed. Results are presented as ORs and 95% confidence intervals (CI). During the interviews, spontaneous comments and open-ended data were written out in full in the respondents’ own words. This material was then read through and analysed together by the main researchers at the office. Quotation contents analysis relied on the agreement of the main researchers [26].

## Results

### Participants, smoking behaviour and cessation support

Table 1 describes general characteristics of the sample and smoking behavior by age and gender. Overall smoking prevalence was 43.6% (95% CI-34.4–46.6). When questioned about other colleague’s smoking behaviour, 43.8% of those who shared a taxi with a colleague answered that their colleague was a smoker and 7.3% did not know. Smoking behaviour was significantly associated with shift-work (smokers: dayshift-30.4%; nightshift- 67.6%; both-41.8%; p < 0.001). Current smokers reported smoking on average 25.5 ± 3.0 cigarettes per day (median = 20); and ex-smokers 37.5 ± 5.2 (median = 35), p < 0.001. Of the smokers, 52.3% reported they did not want to quit. All ex-smokers,except one, had quit “cold turkey”, usually after a tobacco-related illness or life threatening event. Two participants reported having used over the counter NRT. None of the participants had ever attended a smoking prevention session addressing the ban, SHS health hazards, or cessation support.

**Table 1:** General characteristics of the sample and smoking behavior by age and gender *

**Gender**	**n**	**%**						
Male	249	99.6						
Female	1	0.4						
**Nationality**								
Portuguese	246	98.4						
Non-Portuguese	4	1.6						
**Work-shift**								
Day	115	46.0						
Night	68	27.2						
Both	67	26.8						
Education**								
4–6	135	54.0						
7–9	65	26.0						
10–11	26	10.4						
12	23	9.2						
Higher Education	1	0.4						
**Taxi sharing with colleague**								
Yes	178	71.2						
No	72	28.8						
**Total**	250	100						
**Age-Group**	**Smokers**		**Ex-smokers**		**Never-smokers**		**Total**	
	**n**	**%**	**n**	**%**	**n**	**%**	**n**	**%**
**25–44**	30	60.0	9	18.0	11	22.0	50	100
**45–54**	42	51.2	23	28.0	17	20.7	82	100
**55–64**	30	35.3	36	42.4	19	22.4	85	100
**65–76**	7	21.9	14	43.8	11	34.4	32	100
**Total**	109	43.8	82	32.9	58	23.3	249	100
**Females**								
**45–54**	0	0.0	0	0.0	1	100.0	1	100
**Total**	**109**	**43.6**	**82**	**32.8**	**59**	**23.6**	**250**	**100**

* p = 0.005. ** Education in schooling years. In Portugal, since 1986, compulsory education is 9 years. Before 1986 it was 4–6 years.

### SFP awareness and support

All taxis displayed the required signs, concerning the legal ban. Table 2 shows ban support and factors associated with this behaviour. Most participants (82.8%) approved smoking bans in indoor public places. In addition, of those who lived in their own house or owned a car, 55.6% reported a smoke-free (SF) home, and 53.6% a smoke-free car. Support for the ban was significantly associated with smoking status and private smoking restrictions. Day-shift participants reported stronger ban support. In addition, those who indicated health protection as the main reason for the ban reported the strongest ban agreement. Heavy smokers reported less positive attitudes, compared to lighter smokers. Education did not influence ban support (data not shown). MLR showed that being a non-smoker predicted strong ban support, followed by reporting health believes and by reporting a smoke-free car. Table 3 presents self-reported main reasons for the understanding of the ban.

**Table 2:** Ban support and associated factors

***Ban support***		**Strongly agree/agree**		**Strongly disagree/disagree**		**Total**		**Crude OR**	**95%CI**	**p value**	**Adjusted OR**	**95%CI**	**p value**	**MLR (Strong ban support)**
		**n**	**%**	**n**	**%**	**n**	**%**							
**Smoking behaviour**	N-Smokers	133	94.3	8	5.7	141	100	7.86	3.47–17.84	<0.001	6.27	3.05–12.86	<0.001	**Model explanation**
**Ref**	Smokers	74	67.9	35	32.1	109	100							77.8%
	Total	207	82.8	43	17.2	250	100							
Smoke-free Home *	Yes	117	90.7	12	9.3	129	100	3.82	1.84–7.95	<0.001	*ns*			**Hosmer and Lemeshow test**
**Ref**	No	74	71.8	29	28.2	103	100							p = 0.296
	Total	191	82.3	41	17.7	232	100							
Smoke-free Car **	Yes	115	95.8	5	4.2	120	100	11.17	4.17–29.91	<0.001	2.39	1.16–4.90	0.018	**Model p value**
**Ref**	No	70	67.3	34	32.7	104	100							<0.001
	Total	185	82.6	39	17.4	224	100							
**Cigarettes per day**	≤20	19	45.2	23	54.8	42	100	2.63	1.15–6.02	0.20	*ns*			
**Ref**	>20	16	23.9	51	76.1	67	100							
	Total	35	32.1	74	67.9	109	100							
***Strong ban support***		**Yes**			**No**			**Crude OR**	**95%CI**	**p value**	**Adjusted OR**	**95%CI**	**p value**	
		**n**	%*	**n**	**%**	**n**	**%**							
**Shift work**	Day	72	62.6	43	37.4	115	100	3.50	1.86–6.59	<0.001	*ns*			
**Ref**	Night	22	32.4	46	67.6	68	100							
	Total	94	51.4	89	48.6	183	100							
**Health beliefs**	Yes	78	73.6	28	26.4	106	100	4.38	2.53–7.56	<0.001	3.71	1.85–7.43	<0.001	
**Ref**	No	56	38.9	88	61.1	144	100							
	Total	134	53.6	116	46.4	250	100							

*OR* Odds ratio, *MLR* Multiple logistic regression, *Ref* Reference category for comparison; ns: non-significant; * not-applicable: “I live in a rented room” (n = 18); ** not-applicable “I do not have a private car” (n = 26). Adjusted OR derived from MLR model adjusted for driver’s smoking behaviour, Smoke-free car, Smoke-free home, tobacco consumption, shift work, health believes.

**Table 3:** Self-reported main reasons for the understanding of the ban

**Understanding of the ban**	**n**	**%**
· Health/To avoid Diseases	98	39.2
· Respect non-smokers	92	36.8
· To reduce consumption	6	2.4
· Health + Respect non-smokers	8	3.2
· Do not know	31	12.4
· Environmental and pollution control	5	2.0
· Others*	10	4.0
**Total**	250	100.0

*Other reasons: EU imposition, political reasons, “fashion”, “fundamentalism”.

### Ban compliance and enforcement

Tables 4 and 5 present self-enforcement and compliance respectively, and factors associated with this behaviour. Of the smokers, 66.1% admitted smoking in their taxi. When questioned about other colleague’s smoking behaviour, 71.0% of those drivers that shared a taxi with a smoking colleague reported that colleagues that smoked did so inside their taxis (24.4% did not know). 84.8% of the drivers reported that clients still asked to smoke in their taxis (9.6% often; 30.4% sometimes; 44.8% seldom; 15.2% never); 16.8% allowed clients to smoke in their vehicles. Prior to the ban this value was 76.9% (p < 0.001). The major reason for not allowing smoking was the legal ban and associated fines (71.2%). See Table 6. Allowing smoking in private settings was significantly more frequent than inside taxis (p < 0.001). Stale smoke smells were detected in 37.6% of the cars and were significantly associated with the driver being a smoker (p < 0.001), or having a colleague who smoked (p < 0.001), and reporting smoking in the taxi (p < 0.001). Prior to stopping the taxi for the service, researchers observed 5 participants smoking inside the taxi. Another participant smoked during the taxi service. This was the only vehicle in which ashes were observed. In total, we observed 6 (2.4%) participants smoking in the taxi. None of the taxi drivers did ever receive a fine for non-compliance or was stopped by the police. Self-enforcement was reported more often than compliance (p < 0.001), even among smokers (p = 0.001). Ban support was significantly associated with self-enforcement, but not with compliance. Smokers more frequently allowed clients to smoke in their taxi (29.4%) than ex-smokers (4.9%) or never smokers (10.2%), (p < 0.001). The same trend was reported by younger taxi drivers. Participants that reported not allowing smoking prior the ban also reported consistent self-enforcement. Moreover, having a SF car was associated with consistent self-enforcement and compliance. Finally, drivers who reported smoking in the taxi allowed clients to smoke more often. Education did not influence self-enforcement, or compliance (data not shown). MLR showed that being a non-smoker and reporting not allowing smoking prior the ban predicted self-enforcement. Heavy smokers reported smoking in the car more often than lighter smokers. Night-shift participants also reported the same behaviour. Compliance was also significantly associated with reporting a smoke-free car, not allowing smoking prior the ban and reporting health protection as the main reason for the ban. MRL showed that being a heavy smoker, working at night, and allowing smoking prior the ban predicted non-compliance.

**Table 4:** Self-enforcement and associated factors

***Self-enforcement***		**Yes**		**No**		**Total**		**Crude OR**	**95%CI**	**p value**	**Adjusted OR**	**95%CI**	**p value**	**MLR**
		**n**	**%**	**n**	**%**	**n**	**%**							
**Smoking behaviour**	N-Smokers	131	92.9	10	7.1	141	100	5.44	2.54–11.69	<0.001	3.94	1.77–8.75	0.001	**Model explanation**
**Ref**	Smokers	77	70.6	32	29.4	109	100							82.9%
	Total	208	83.2.	42	16.8	250	100							
**Smoking Drivers’ Compliance**	Yes	35	94.6	2	5.4	37	100	12.50	2.79–56.02	<0.001	ns			**Hosmer &Lemeshow test**
**Ref**	No	42	58.3	30	41.7	72	100							P value = 0.264
	Total	77	70.6	32	29.4	109	100							
Smoke-free Car *	Yes	111	92.5.	9	7.5	120	100	4.54	2.03–10.17	<0.001	ns			**Model p value**
**Ref**	No	76	73.1	28	26.9	104	100							<0.001
	Total	187	83.5	37	16.5	224	100							
Allowing smoking prior to the ban**	No	48	96.0	2	4.00	50	100	6.41	1.49–27.69	0.005	4.79	1.08–21.16	0.039	
**Ref**	Yes	131	78.9	35	21.1	166	100							
	Total	179	82.9	37	17.1	216	100							
**Age**	≥55	104	88.9	13	11.1	117	100	2.23	1.10–4.53	0.024	ns			
**Ref**	<55	104	78.2	29	21.8	133	100							
	Total	208	83.2	42	16.8	250	100							
**Ban support**	Yes	179	86.5	28	13.5	207	100	3.09	1.46–6.55	0.002	ns			
**Ref**	No	29	67.4	14	32.6	43	100							
	Total	208	83.2	42	16.8	250	100							

*OR* Odds ratio, *MLR* Multiple logistic regression, *Ref* Reference category for comparison; ns: non-significant; *not-applicable: “I do not have a car” (n = 26); **not-applicable: “I did not work as a taxi driver prior the ban” (n = 34); Adjusted OR derives from MLR adjusted for drivers’ smoking behaviour, allowing smoking prior to the ban, smoke-free car, age, ban support.

**Table 5:** Compliance and associated factors

***Compliance***		**Yes**		**No**		**Total**		**Crude OR**	**95%CI**	**p**	**Adjusted OR**	**95%CI**	**p**	**MLR**
		**n**	**%**	**n**	**%**	**n**	**%**							
**Cigarettes per day**	>20	3	7.1	39	92.9	42	100	0.08	0.02–0.27	<0.001	0.045	0.01–0.38	0.004	**Model explanation**
**Ref**	≤20	34	50.7	33	49.3	67	100							77.6%
	Total	37	33.9	72	66.1	109	100							
Allowing smoking* prior to the ban	Yes	21	25.9	60	74.1	81	100	0.18	0.05–0.64	0.004	0.15	0.02–0.88	0.036	**Hosmer and Lemeshow test**
**Ref**	No	8	66.7	4	33.3	12	100							p = 0.950
	Total	29	31.2	64	68.8	93	100							
Smoke-free car**	Yes	13	56.5	10	43.5	23	100	4.12	1.55–10.97	0.003	ns			**Model p value**
**Ref**	No	18	24.0	57	76.0	75	100							<0.001
	Total	31	31.6	67	68.4	98	100							
**Shift work**	Night	11	23.9	35	76.1	46	100	0.30	0.12–0.77	0.011	0.20	0.05–0.79	0.021	
**Ref**	Day	18	51.4	17	48.6	35	100							
	Total	29	36.3	52	63.8	81	100							
**Health beliefs**	Yes	16	48.5	17	51.5	33	100	2.47	1.06–5.76	0.035	ns			
**Ref**	No	21	27.6	55	72.4	76	100							
	Total	37	33.9	72	66.1	109	100							

*OR* Odds ratio, *MLR* Multiple logistic regression, *Ref* Reference category for comparison; ns: non-significant; **not-applicable: “I did not work as a taxi driver prior the ban” (n = 16); *not-applicable: “I do not have a car” (n = 11); adjusted OR derived from MLR adjusted for driver’s tobacco consumption, allowing smoking prior to the ban, Smoke-free car, shift work, health believes.

**Table 6:** Reasons for not allowing clients smoking in the taxi

**Before the ban**	**n**	**%**
SHS annoyance	19	36.0
To avoid diseases/own health	11	26.0
SHS annoys clients & damages the car	19	36.0
It is forbidden	1	2.0
**Total**	**50**	**100.0**
**After the ban**	**n**	**%**
SHS annoyance & legal ban	29	13.9
To avoid diseases/own health & legal ban	16	7.7
SHS annoys clients, damages the car & legal ban	15	7.2
The legal ban and associated fines	148	71.2
**Total**	**208**	**100.0**

### Quotation contents analysis

Relevant recurrent themes included: “It’s very difficult to quit… and … to abstain from smoking in the taxi…due to work-related stress”. “We’re aware that many colleagues are heavy smokers…and that they frequently smoke in their taxis… when they’re alone… but they’re not fined, so they continue to smoke”. “The ban cannot be effective because of poor enforcement and lack of sanctions”, “the authorities are at fault cause they don’t apply the fines…”, “it was difficult to forbid smoking before the ban… we couldn’t risk losing clients.. SFPs are a good thing indeed”. “Usually I don’t allow smoking in the taxi, but in special occasions, I do: on longer trips…at night… when enforcement is less probable …“when clients are very nervous or disturbed… we shouldn’t displease our clients”. “Tobacco smoke annoys clients … and ruins the upholstery of the car” “Clients ask more often to smoke at night… when they’ve drunk alcohol, or young people ask to smoke quite often”. We also observed that a smoke-free home depends specially on the family environment (others smokers, pressure from spouses, children or asthmatic relatives).

## Discussion

All taxi drivers were aware of the smoking ban. Most of them reported ban support, understood this public health policy, and did not allow clients to smoke. In addition, a considerable proportion of participants reported smoking restrictions in their own private settings, although less often than mandatory legal restrictions. Nevertheless, while most taxi drivers approve the ban, the majority of smokers do not comply with it. In addition, most participants reported that clients still asked to smoke inside the taxi, and a significant minority allowed clients to smoke. Stale smoke smells were detected in a significant number of vehicles. Moreover, enforcement by the authorities was never reported. Our study shows that the majority of taxi drivers support comprehensive SFPs, highlighting that these policies are indeed highly popular, even among smokers, and in pro-smoking environments [3-5,9,10,12]. However, a significant proportion of participants admitted smoking in the car, as in other studies [12,13,28,29]. Furthermore, compliance was strongly predicted by smoking behaviour and not by attitudes to SFPs, as previous research has shown in other enclosed settings [10,12]. While the main reason for not allowing clients smoke in the taxi was the legal ban and associated fines, compliance was influenced by smoking behaviour, work-shift and participants’ norms prior the ban. Recent observational studies have estimated prevalence of tobacco consumption and SHS exposure in vehicles. Prevalence was significantly lower than our study, and also lower in taxis than other commercial vehicles or private cars. However, observational studies usually report less SHS exposure than questionnaire-based surveys [24,30-32]. Moreover, our findings show that usually taxi drivers smoke in the taxi when they are alone. In year 2006, 57% of the Portuguese smokers declared smoking in the car [28]. A recent population-based study in Portugal observed that 64% of daily smokers reported smoking in their private cars [29]. In our study, taxi drivers reported smoking in the car about the same frequency. Vehicles that correspond to workplaces or public transports are required to be smoke-free under the Portuguese law. It is also worth noting that participants reported that young people often ask to smoke in the taxis. All these findings suggest that social smoking norms suffered little change after the ban. In addition, we may conclude that the ban is not effective in this particular setting. Quotation contents analysis suggests that pressure from clients also influences compliance. This may contribute to further breaches associated with partial bans and economic recession. Self-reported reasons for not allowing smoking included SHS annoyance, health beliefs, and work-related beliefs and rules. Ban support was influenced by SHS health beliefs, and was significantly associated with self-enforcement. Self-enforcement dramatically improved after the ban came into force, and was more frequently reported than compliance, even among smokers. All these findings are consistent with other studies and point out the importance of public health media campaigns, to promote public awareness and acceptance, educating about SHS health hazards and empowering citizens to enforce the law [3-5,9,12,27,33,34]. Health hazards concerning SHS exposure in vehicles should be emphasised. In addition, our study highlights the need to promote smoke-free cars and enforce bans in taxis and other commercial vehicles, in line with recent research [13,24,30-32]. This communication strategy should take into account normative beliefs and workplace rules and beliefs [33,34], as enforcement in vehicles may be difficult and less feasible [35]. Regarding education, our sample was rather homogeneous, revealing very low education levels. This may explain why education did influence neither ban support, nor compliance. Moreover, Hitchman et al have also observed that smoking in cars was not related to education [13]. There is evidence from the literature that ban support increases over time, after the implementation of a smoking ban. In addition, compliance may worsen over time when a partial and poorly enforced ban is implemented [2,7,19,36]. Thus we expected to observe some change over time, concerning support and compliance with the ban. Nevertheless, we did not observed any effect over the study time period. This may be explained as our study has begun one year after the ban came into force. Generally, the major changes occur in the first year of the implementation [2]. In addition, we may admit that this effect happened more often in other settings such us hospitality venues, and especially after the recession period [19,22]. When questioned about their understanding of the ban, one of the main reasons was: “respect for non-smokers”. This finding is rather peculiar and may be influenced by no-smoking signs. In Portugal, no-smoking signs required by law do not include “no smoking” or “smoking is against the law”, instead they indicate “smokers” (blue signs) or “non-smokers” (red signs) [37]. This undermines the message of law breaching, while splits smokers and non-smokers, and may cause conflicts and arguments for “smokers’ rights”. Smoking prevalence was high. Most of the smokers reported being heavy smokers and did not want to quit, which indicates high tobacco dependence [38]. Nevertheless, participants did not report any cessation support by occupational health services. Our findings suggest that there is a need for comprehensive tobacco control policies targeting workplaces and specifically high risk groups, such as heavy smokers, low socio-economic status, and night-shift and vehicles workers. Partials bans do not target high risk groups in vulnerable settings. Workplace-based tobacco control programmes should become a priority and might contribute to ban support and compliance, including self-enforcement. Occupational health and human resources services should play a major role regarding SFP awareness and cessation. In general population, non-smokers are the majority. Promotion of citizens’ enforcement, and non-smokers’ assertiveness, through social environments and networks, should be encouraged [27,34]. Our findings show that a partial and poorly-enforced ban is vulnerable to breaches. The Portuguese smoking ban implementation was followed by popular media debates using “pro & cons” discussion style, instead of SFP evidence-based arguments. Public health education campaigns were absent. Since its implementation, exemptions and breaches, and ineffective enforcement were announced in the media. Important public figures, who should be role models, including the chief of the law enforcement body, and the Prime Minister, were involved in smoking ban public breaches and were not fined. The hospitality industry claimed for exemptions. An eminent law professor did argue against these exemptions, emphasizing that they could not protect all citizens, violating the general principle of health protection of Portuguese law. Following a polemic “media war”, health authorities finally accepted these exemptions [19,39-41]. All these events definitely menaced the credibility of the smoking ban. SFP legislation is necessary to allow enforcement and to promote social acceptance. However, as highlighted by Spinney, 2007, p. 1507 [36], SFPs “will fail if they do not send out a clear message from the start”. In addition, SFPs may turn backwards over time, if they are not enforced and publicized [2,36]. A partial ban does not protect all citizens from SHS exposure and may widen social and health inequalities. In addition, partial bans may also undermine social norm change [8,12,42]. Moreover, without timely-sustained education campaigns, promoting SFP awareness, and citizens’ law enforcement, laws may not warrant compliance. A partial ban is, therefore, less cost-effective and more difficult to comply with than a comprehensive ban, and may raise enforcement costs [2,9]. These should be key messages for policy makers.

### Strengths and limitations

Few studies have addressed enforcement and compliance with partials smoking bans, especially in taxis or other commercial vehicles. The study information was collected from a relative large number of interviews, and participation rate was high. Researchers were trained to minimize interviewer bias and approached the participants in their natural environment, as clients. Empathic relationship between interviewers and participants was easily achieved. However interviewer, observer, and social desirability biases cannot be excluded. The cross-sectional nature of the study does not allow causal inferences. Purposive sampling limits generalization to different populations. Sampling selection bias should also be considered.

## Conclusions

Despite the strong ban support observed, high smoking prevalence and poor enforcement contribute to low compliance. The findings also suggest low compliance among night-shift and vehicle workers. This study clearly demonstrates that a partial and poorly-enforced ban is vulnerable to breaches, and highlights the need for clear and strong policies.

## Abbreviations

CI: Confidence intervals; MLR: Multiple logistic regression model; NRT: Nicotine replacement therapy; NS: Non significant; OR: Odds ratio; REF: Reference; SF: Smoke-free; SFPs: Smoke-free policies; SHS: Second-hand smoke.

## Competing interests

The authors declare that they have no competing interests.

## Authors’ contributions

SBR conceived the study and the study design, coordinated and participated in the field work, performed the statistical analysis, analysed and interpreted the data, and wrote the manuscript. JMC participated in the study design and in the field work, analysed and interpreted the data and contributed to the drafting of the manuscript. PA coordinated the statistical analysis, analysed and interpreted the data and critically reviewed the paper. MCB analysed and interpreted the data and critically reviewed the paper. All authors contributed, read and approved the final manuscript.

## Pre-publication history

The pre-publication history for this paper can be accessed here:

http://www.biomedcentral.com/1471-2458/13/134/prepub
